# Correlated Color Temperature Affects Image-Based Color Analysis of Chicken Breast and Drumstick: Implications for Using 6500 K as a Reference

**DOI:** 10.3390/foods15152602

**Published:** 2026-07-24

**Authors:** Alper Güngören, Gülşah Güngören

**Affiliations:** 1 Department of Food Hygiene and Technology, Faculty of Veterinary Medicine, Kastamonu University, 37150 Kastamonu, Türkiye; 2 Department of Animal Science, Faculty of Veterinary Medicine, Kastamonu University, 37150 Kastamonu, Türkiye

**Keywords:** correlated color temperature, breast meat, drumstick, computer vision, CIELAB, color measurement, standardization, meat color

## Abstract

Correlated color temperature (CCT) can substantially influence image-based color measurements, though its effects vary with the optical and physical properties of the meat surface being evaluated. This study investigated the effects of CCT on the image-based color properties of skinless chicken breast and skin-on drumstick samples. It evaluated the suitability of 6500 K as a reference condition. Seventeen skinless breast and seventeen skin-on drumstick samples were repeatedly photographed under 13 CCT conditions ranging from 2500 to 8500 K at 500 K intervals, producing 442 observations. Camera settings, imaging geometry, illuminance, and sample position were maintained constant. Image-derived L*, a*, and b* values were obtained using the Fiji freeware program, and chroma (C*), whiteness index (WI), yellowness index (YI), browning index (BI), and total color difference (ΔE*ab) relative to 6500 K were calculated. Data were analyzed using linear mixed models, and the common variation in L*, a*, and b* was summarized by principal component analysis. Sample type, CCT, and their interaction significantly affected all evaluated parameters (*p* < 0.001). Increasing CCT generally increased L* and WI, while a*, b*, C*, YI, and BI decreased markedly, indicating a transition toward a lighter and less chromatic image-derived appearance. The largest deviations from the 6500 K reference occurred at 2500 K, with ΔE*ab values of 42.86 and 43.57 for breast and drumstick samples, respectively. Even at 6000 K, only 500 K below the reference condition, ΔE*ab exceeded 4.0 in both breast and drumstick samples, indicating visually perceptible color differences. Visually relevant differences also persisted above 6500 K. The first principal component explained 86.89% of the total variance and described the coordinated increase in lightness and reduction in redness and yellowness with increasing CCT. These findings demonstrate that CCT is a major source of systematic variability in image-based poultry color analysis workflow; therefore, 6500 K should not be treated as a universal reference unless the complete illumination, camera, calibration, and image-processing protocol is standardized and validated for the specific poultry surface being examined.

## 1. Introduction

Color is one of the most important quality attributes influencing consumer perception and acceptance of poultry meat and meat products. It is commonly associated with freshness, storage conditions, and overall product quality and may therefore strongly affect purchasing decisions [1,2,3]. Although color does not independently determine the safety or nutritional quality of meat, an unusual or undesirable appearance may result in product rejection even when other quality characteristics remain acceptable [1,2,4,5]. Objective and reproducible color examination is consequently important for quality control, product development, and the comparison of results among studies [6].

Commercial chicken breast and drumstick products differ in their visible surface characteristics. Skinless breast presents a predominantly muscle surface, whereas retail drumsticks are commonly marketed with the skin intact, resulting in a composite optical surface influenced by skin, subcutaneous fat, connective tissue, and underlying muscle. Breast meat is generally lighter and less pigmented, whereas drumstick meat has a darker and more reddish appearance because of differences in muscle function, fiber composition, vascularization, and myoglobin content [7,8]. These tissue-specific properties may also influence how the meat surface interacts with incident light. Therefore, an illumination condition that provides suitable Image processing system (IPS)-based color information for breast meat may not necessarily produce the same answer in drumstick meat. The simultaneous evaluation of both product types is particularly relevant when setting standardized imaging protocols for poultry meat.

Meat color is traditionally evaluated using sensory panels, colorimeters, or spectrophotometers. Sensory assessment provides direct information on visually perceived color but can be complex and time consuming, and its reproducibility may be affected by panelist-related factors, visual sensitivity, tiredness, lighting, and other viewing conditions [4,6,9]. Instrumental colorimeters provide rapid and objective measurements; however, their relatively small measurement area, typically covering only approximately 2–5 cm^2^, represents only a limited portion of heterogeneous meat surfaces [10,11,12]. Consequently, the number of measurements should be increased and readings should be obtained from multiple representative locations to reduce the influence of local color heterogeneity and obtain a more reliable estimate of the overall surface color. Nevertheless, meat is not a completely opaque and diffusely reflecting surface; instead, it behaves as a partially translucent and optically cloudy matrix. Incident light can penetrate beneath the meat surface and undergo wavelength-dependent absorption by chromophores such as myoglobin, together with multiple scattering, reflection, and refraction within the tissue. Consequently, local variation in pigmentation, intramuscular fat and connective tissue distribution, surface texture, and light scattering and reflection may influence the recorded color values [6,10,13,14,15]. Image processing systems (IPSs) offer an alternative approach by evaluating a larger portion of the sample surface and providing spatially representative color information. Digital images can be transformed into the CIELAB color space, in which L*, a*, and b* describe lightness, red–green position, and yellow–blue position, respectively. Derived parameters and indices can additionally be used to characterize color saturation (Chroma), whiteness, yellowness, browning, and total color difference [1,14,15,16].

Despite these advantages, the reliability of IPS-based color analysis depends strongly on the standardization of image acquisition conditions. Camera settings, viewing geometry, background, sample position, exposure, light intensity, spectral composition, and correlated color temperature (CCT) can all alter the recorded pixel values. CCT, expressed in kelvin (K), describes the apparent warm or cool color of a light source by relating its chromaticity to that of an ideal black-body radiator. Lower CCT values generally correspond to warmer, yellow–red illumination, whereas higher CCT values correspond to cooler, white–blue illumination. Changes in CCT also alter the relative contribution of long- and short-wavelength light. Consequently, the same meat sample may produce different CIELAB values when photographed under different CCT conditions, even when the camera, sample, and imaging geometry remain unchanged [4,10,14,17].

CIE standard illuminant D65 is a mathematically defined daylight illuminant with a specified spectral power distribution and a correlated color temperature of approximately 6500 K, and it is widely used as standard illuminant in instrumental and IPS-based color measurements [17,18,19,20,21,22]. However, a physical LED source adjusted to a nominal CCT of 6500 K is not necessarily spectrally equivalent to D65. Accordingly, the 6500 K LED setting used in the present study was selected as a practically relevant, device-specific reference condition rather than as a physical reproduction of D65. Its widespread use does not necessarily demonstrate that it is an optimal or universal reference for all meat types. Previous IPS research on red meat showed that changing CCT substantially affected the recorded color parameters and indicated that illumination conditions should be selected according to the optical characteristics of the evaluated material [4].

Poultry breast and drumstick meat may differ not only from red meat but also from each other in lightness, pigmentation, and spectral reflectance. Nevertheless, information regarding their comparative responses across a broad CCT range remains limited. To our knowledge, it has not been clearly established whether a 6500 K condition provides a suitable common reference for IPS color measurements of both products.

Accordingly, the present study aimed to determine the effects of CCT conditions ranging from 2500 to 8500 K on the IPS color characteristics within a specific camera-LED-Fiji workflow applied to skinless chicken breast and skin-on drumstick surfaces. The specific objectives were to evaluate changes in the primary CIELAB coordinates and derived color indices, determine whether the two samples responded differently to CCT, quantify total color differences relative to the 6500 K reference condition, and summarize the joint variation in L*, a*, and b* values using principal component analysis. The findings are expected to contribute to the standardization of illumination conditions and the development of more reliable image-based protocols for poultry meat color assessment.

## 2. Materials and Methods

### 2.1. Meat Samples and Experimental Design

The study comprised 442 IPS-based observations obtained from 34 poultry meat samples, including 17 breast and 17 drumstick samples. Fresh chicken breast and drumstick samples produced by Banvit/BFR (Balıkesir, Turkey), all within the early stage of their stated shelf life (from the same production batch), were obtained from a local retail market (Kastamonu, Turkiye). The samples were supplied in overwrapped trays. Breast samples were evaluated without skin, whereas drumstick samples were evaluated with the skin intact because these conditions reflected their typical commercial presentation at retail. Accordingly, the study was designed to compare the image-derived color responses of commercially relevant poultry product surfaces rather than to isolate intrinsic differences between breast and drumstick muscle samples. The samples were brought to the laboratory in a cold chain (4 ± 1 °C) and quickly assigned a unique identification number. Samples were stored under refrigerated conditions at 6 ± 1 °C and were removed individually from storage only during image acquisition and analysis. The CCT conditions were applied sequentially in ascending order, with all samples photographed first at 2500 K and subsequently at 500 K increments up to 8500 K. The required CCT was set before each imaging series, while the camera position, imaging chamber, illumination geometry, and camera settings remained unchanged. Samples awaiting imaging were maintained under refrigeration and were removed individually only for photography. Each sample was placed in the imaging chamber and photographed within approximately 5–10 s. Image acquisition commenced immediately after arrival at the laboratory, and the complete imaging procedure was completed within approximately 4 h. After each image was acquired, the sample was immediately returned to refrigerated storage until the next CCT condition was applied.

Each biological sample was evaluated repeatedly under 13 correlated color temperature (CCT) conditions, generating 221 observations per product type and 442 observations in total (34 samples × 13 CCT levels). This repeated-measures design enabled each sample to serve as its own control across illumination conditions, thereby reducing the influence of between sample biological variability.

### 2.2. IPS Setup, Image Acquisition, and Illumination Conditions

**Figure 1:**
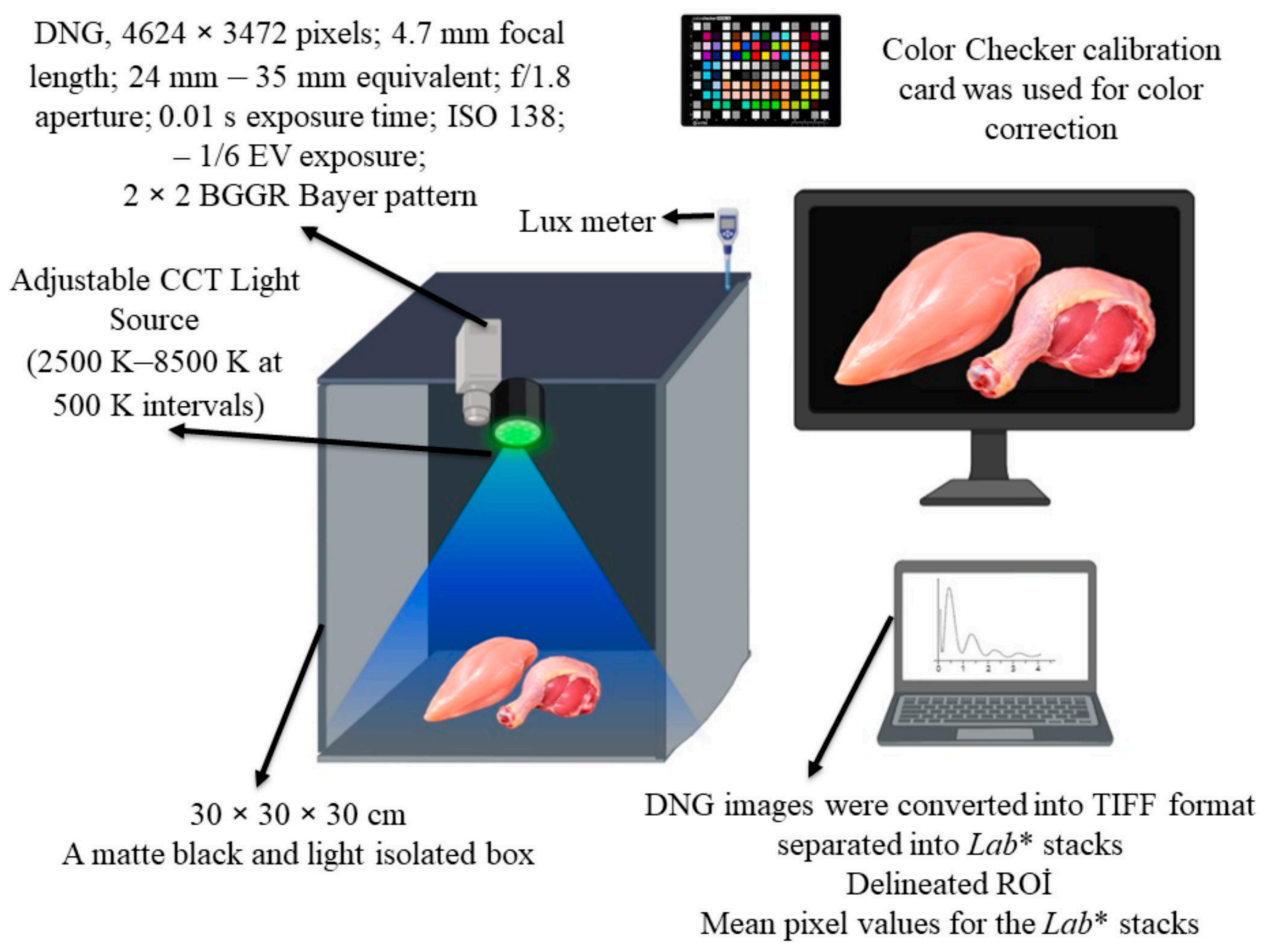
Schematic representation of the image processing system used for chicken breast and drumstick meat imaging, including the light-isolated imaging box, adjustable CCT light source, camera settings, illuminance measurement, color calibration, and subsequent image-processing workflow.

The image processing system (IPS) consisted of four main components: a custom built-matte black, light-isolated imaging chamber measuring 30 × 30 × 30 cm, a camera equipped with a complementary metal oxide semiconductor (CMOS) image sensor (Xiaomi 23049PCD8G, POCO, Beijing, China), a controllable LED light source with high color rendering index > 95 (VL49RGB, ULANZI, Hong Kong, China), and a computer for image transfer and analysis (Figure 1).

Image acquisition was performed inside a custom-built, matte black, light-isolated imaging chamber. The matte black interior surfaces and background were used to minimize ambient light contamination, stray reflections, and background-induced color interference [4]. Each meat sample was placed in a transparent plastic tray at the same fixed position on the dark background. Images were acquired using the Open Camera application (https://www.opencamera.org.uk/, accessed on 1 may 2026) installed on a smartphone (Xiaomi, 23049PCD8G, POCO, Beijing, China), which was mounted vertically above and 90° to the sample surface. The camera was positioned vertically at a fixed distance of 30 cm above the sample and connected to an external monitor (LG-29UM68-P, Seoul, Republic of Korea) for visualization. Illuminance at the sample surface was measured using a lux meter (Arduino-based DIY model) and maintained at approximately constant levels under all CCT conditions. A Calibrite Color Checker target was used during the preliminary setup under the 6500 K LED condition to verify the imaging arrangement and establish the fixed camera settings. It was not used to generate a camera color profile or to apply color correction to the images. White balance, exposure time, ISO sensitivity, focus, and exposure compensation were fixed before experimental imaging and remained unchanged throughout the acquisition of all images. All images were captured in Digital Negative (DNG) raw format at a resolution of 4624 × 3472 pixels. The camera operated with a focal length of 4.7 mm, corresponding to a 35 mm equivalent focal length of 24 mm, and an aperture of f/1.8. Exposure time was fixed at 0.01 s (approximately 1/99 s), ISO sensitivity was fixed at 138, and exposure compensation was set to −1/6 EV. Flash was disabled, and average metering was used throughout image acquisition. Focus was fixed before image acquisition and remained unchanged for all images. Exposure, focal length, focus, camera position, imaging distance, sample position, and illumination geometry were maintained constant throughout the experiment to ensure standardized image acquisition. The raw images contained single sample per pixel color filter array data arranged in a 2 × 2 BGGR Bayer pattern.

**Figure 2:**
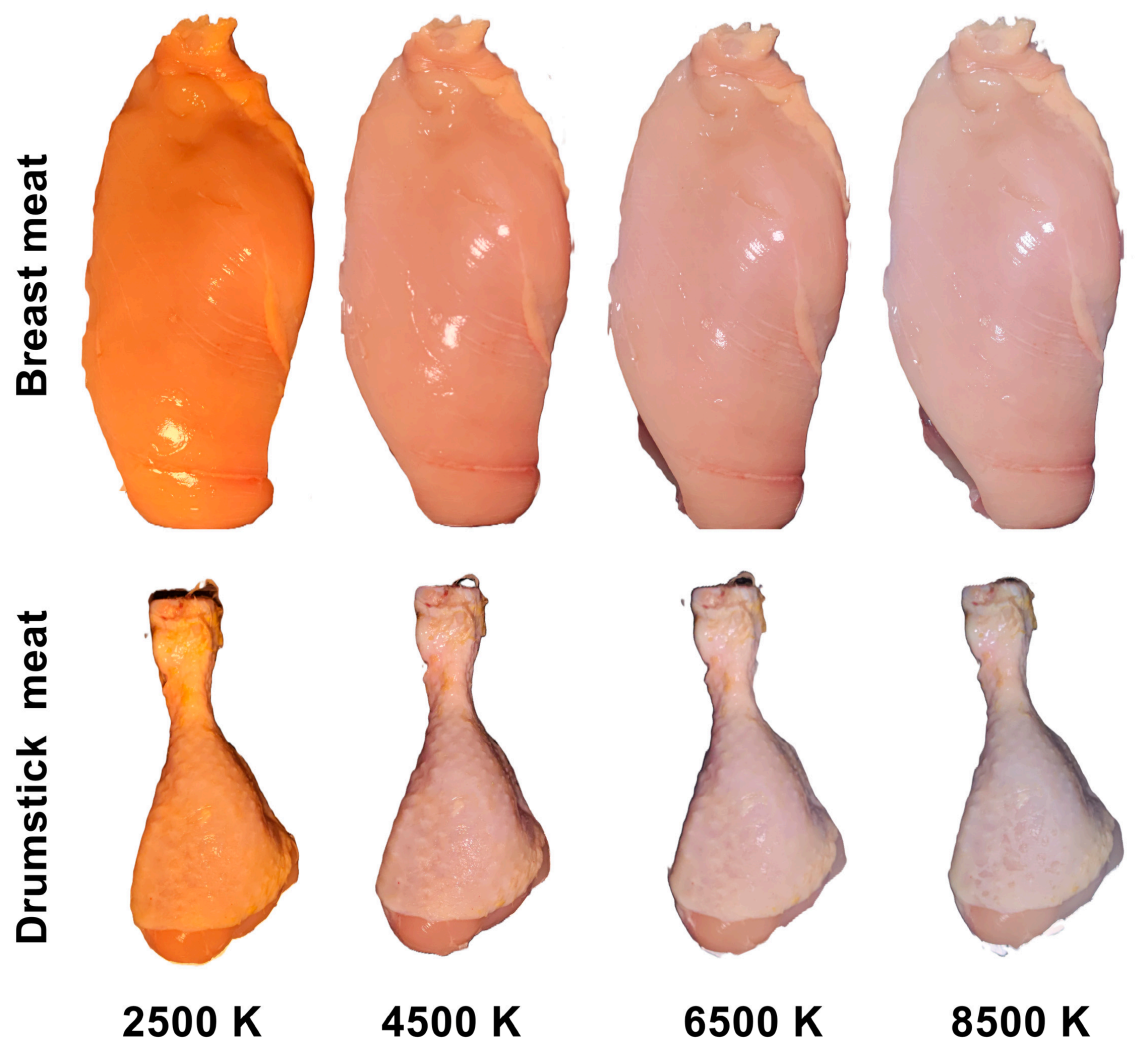
Representative images of chicken breast and drumstick meat acquired under selected correlated color temperature conditions (2500, 4500, 6500, and 8500 K). The same biological sample of each product was photographed under all illumination conditions while camera settings, imaging geometry, sample position, and light-source position were maintained constant. The 6500 K condition was used as the reference illumination.

Images were acquired under 13 CCT conditions ranging from 2500 to 8500 K at 500 K intervals: 2500, 3000, 3500, 4000, 4500, 5000, 5500, 6000, 6500, 7000, 7500, 8000, and 8500 K. The 6500 K reference condition was designated as the reference illumination (Figure 2).

### 2.3. Image Analysis and Color Parameter Calculation

**Figure 3:**
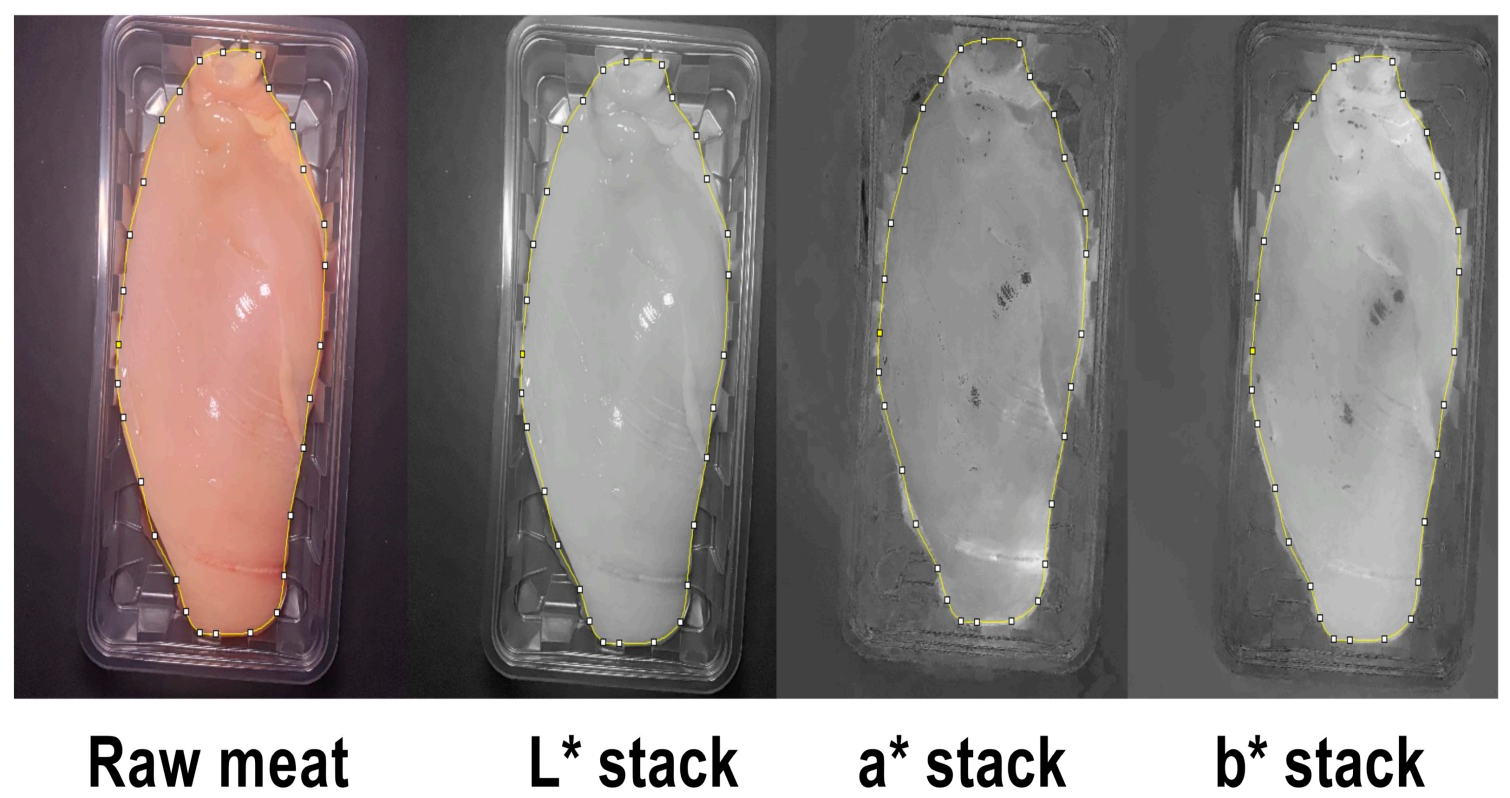
Representative workflow for image-based color extraction from a chicken breast sample. The converted image was separated into L*, a*, and b* stacks in Fiji app, and the meat surface was manually delineated as ROI while excluding the transparent tray and surrounding background. Mean pixel values were calculated within the same region of interest for each stack.

The captured DNG images were converted locally to TIFF format using the WorkinTool Image Converter (V4.9.10.0) installed on a computer. The conversion was performed using the software’s default export settings. No optional resizing, exposure correction, color enhancement, contrast adjustment, sharpening, denoising, saturation adjustment, or other automatic image-processing function was applied. The software did not provide user-accessible controls for output color-profile assignment, gamma correction, or white-balance handling. The resulting TIFF files were subsequently imported into Fiji freeware for image analysis [23]. Color information was extracted in the CIELAB color space. Each image was separated into L*, a*, and b* stacks, and the meat surface was manually delineated as the region of interest (ROI) while excluding the transparent tray and surrounding dark background. The same ROI was applied to the corresponding L*, a*, and b* stacks, and mean pixel values were calculated for each channel. Mean pixel values for the L*, a*, and b* stacks were then calculated over the selected region. Because the same biological sample was photographed repeatedly under different correlated color temperature conditions, color measurements obtained at each CCT were matched within sample. The same ROI was applied to the corresponding Lab* stacks, and mean pixel values were calculated for each stack (Figure 3).

Chroma (C*), whiteness index (WI), yellowness index (YI), browning index (BI), and total color difference (ΔE*ab) were calculated from the image-derived CIELAB values. Hue angle was not reported because it is mathematically derived from a* and b* and became unstable under high-CCT conditions when b* approached zero and the observations crossed the 0°/360° boundary. Therefore, the primary CIELAB coordinates and C* were considered more appropriate for statistical evaluation. For ΔE*ab, the image acquired from the same biological sample at 6500 K was used as the reference condition. The equations used for the calculation of C*, WI, YI, BI, and ΔE*ab are presented below, respectively [1,16].

(1) $$C^{*}=\sqrt{{\left(a^{*}\right)}^{2}+{\left(b^{*}\right)}^{2}}$$

(2) $$WI=100-\sqrt{{\left(100-L^{*}\right)}^{2}+({a^{*}}^{2}+{b^{*}}^{2})}$$

(3) $$YI=\frac{142.86\times b^{*}}{L^{*}}$$

(4) $$BI=\frac{100\times \left(\left(\frac{a^{*}+1.75\times L^{*}}{5.645\times L^{*}+a^{*}-3.012\times b^{*}}\right)-0.31\right)}{0.172}$$

(5) $$\Delta E^{*}ab=\sqrt{{\left(L^{*}-{L^{*}}_{6500}\right)}^{2}{+(a^{*}-{a^{*}}_{6500})}^{2}+({b^{*}-{b^{*}}_{6500})}^{2}}$$

### 2.4. Statistical Analysis

The raw data are provided as Supplementary Materials. The statistical analyses were performed using IBM SPSS Statistics version 27.0 (IBM Corp., Armonk, NY, USA). The experimental design consisted of repeated measurements obtained from 34 biological samples, including 17 chicken breast and 17 drumstick samples, each photographed under 13 correlated color temperature conditions ranging from 2500 to 8500 K. For each dependent variable, including L*, a*, b*, C*, WI, YI, BI, and ΔE*ab, a linear mixed model was fitted to account for the correlation among repeated measurements obtained from the same biological sample. Product type, CCT, and the product × CCT interaction were included as fixed effects. Biological sample was specified as the subject factor, and CCT was defined as the repeated factor. A first-order autoregressive covariance structure, AR(1), was used to model the correlation between successive CCT measurements. Model parameters were estimated using the restricted maximum likelihood method.

The statistical model was expressed as follows;

Y_ijk_ = μ + T_i_ + C_j_ + (T × C)_ij_ + S_k(i)_ + ε_ijk_

where Y_ijk_ represents the dependent color parameter measured in product type i, at CCT level j, and biological sample k; μ is the overall mean; T_i_ is the fixed effect of product type; C_j_ is the fixed effect of CCT; (T × C)_ij_ is the product × CCT interaction; S_k(i)_ is the random effect of the biological sample nested within product type; and εijk is the residual error.

The significance of the fixed effects was evaluated using Type III tests, with denominator degrees of freedom estimated using the Satterthwaite approximation. When a significant product × CCT interaction was detected, the effect of CCT was evaluated separately within each product type, and breast and drumstick values were compared within each CCT condition. Pairwise comparisons were adjusted using the Sidak method [24]. Statistical significance was accepted at *p* < 0.05. For ΔE*ab, the color coordinates obtained from the same biological sample at 6500 K were used as the reference values. Because ΔE*ab was necessarily zero at 6500 K for every biological sample, observations obtained at 6500 K were excluded from the inferential analysis of ΔE*ab. Model assumptions were evaluated by examining standardized residuals, normal probability plots, and plots of residuals against predicted values. In addition, principal component analysis (PCA) was performed on standardized L*, a*, and b* values from all 442 observations using the correlation matrix and principal component extraction without rotation. The component scores were calculated using the regression method. For graphical presentation, the original PC1 regression scores were multiplied by −1 (PC1_reversed_ = −PC1_original_). The component loadings were multiplied by −1 accordingly. This sign transformation changed only the direction of the component axis and did not alter the eigenvalue, explained variance, or relative positions of the observations, and the distributions were visualized using GraphPad Prism version 8.0.

### 2.5. Limitations of the Methods

Several methodological limitations should be considered when interpreting the present findings. First, no spectroradiometric measurements of the SPD of the light source or the spectral reflectance distribution (SRD) of the samples were performed due to the lack of equipment. Instead, a high-CRI (>95) LED light source and controlled illuminance were used to minimize uncontrolled spectral variability [4]. Second, the study evaluated two commercially relevant poultry surfaces obtained from a single production batch: skinless breast meat and skin-on drumsticks. Consequently, the observed differences between sample types represent the combined effects of anatomical origin, skin coverage, subcutaneous fat, connective tissue, surface texture, and optical properties, rather than intrinsic differences between breast and drumstick muscle alone.

## 3. Results

### 3.1. Effects of Product Type and Correlated Color Temperature on CIELAB Color Parameters

The IPS-collected CIELAB color parameters of chicken breast and drumstick meat under different correlated color temperature conditions are presented in Table 1.

The linear mixed model analysis showed significant effects of product type, CCT, and the product × CCT interaction on Lab*, and C* values (*p* < 0.001). The effect of CCT on L* differed between breast and drumstick meat. In breast meat, L* initially decreased from 60.21 at 2500 K to 59.05 at 3000 K and subsequently increased, reaching its highest value of 69.63 at 8500 K. Drumstick meat exhibited higher L* values than breast meat at 2500–4000 K and 5500–7000 K (*p* < 0.05). However, this difference was not significant at 4500, 5000, 7500, or 8000 K (*p* > 0.05), whereas breast meat had a higher L* value than drumstick meat at 8500 K. The maximum L* value for drumstick meat was observed at 7500 K (69.52).

Both a* and b* values progressively decreased as CCT increased, although drumstick b* showed a slight numerical increase between 8000 and 8500 K. The a* value of breast meat declined from 34.43 at 2500 K to 12.10 at 8500 K, while that of drumstick meat decreased from 26.83 to 7.79 over the same CCT range. Breast meat consistently exhibited higher a* values than drumstick meat at every CCT condition (*p* < 0.05). Similarly, b* decreased from 52.72 to 8.19 in breast meat and from 47.40 to 1.63 in drumstick meat. Breast meat also had significantly higher b* values than drumstick meat in all CCT conditions (*p* < 0.05).

Consistent with the changes in a* and b*, C* decreased markedly with increasing CCT. In breast meat, C* declined from 63.00 at 2500 K to 14.74 at 8500 K, whereas in drumstick meat it decreased from 54.48 to 8.14. At every CCT, C* was significantly higher in breast meat than in drumstick meat (*p* < 0.05). These results indicated a progressive reduction in color saturation as illumination shifted from warm to cool CCT conditions.

### 3.2. Derived Color Indices and Total Color Difference

The derived color indices and total color differences relative to the 6500 K reference are presented in Table 2.

Product type, CCT, and their interaction significantly affected WI, YI, and BI (*p* < 0.001; Table 3).

WI increased overall with increasing CCT. In drumstick meat, WI reached its maximum at 7500 K and remained at a similar level thereafter. In breast meat, WI rose from 25.46 at 2500 K to 66.23 at 8500 K. In drumstick meat, WI increased from 35.63 to 66.52. Drumstick meat generally exhibited higher WI values than breast meat, particularly between 2500 and 8000 K, whereas no significant product difference was observed at 8500 K.

YI decreased markedly overall as CCT increased, although drumstick YI showed a slight increase between 8000 and 8500 K. Breast meat YI declined from 125.09 at 2500 K to 16.79 at 8500 K, while drumstick meat YI decreased from 103.01 to 3.49. Breast meat had significantly higher YI values than drumstick meat at every CCT. Product type, CCT, and their interaction significantly affected BI (*p* < 0.001). BI decreased markedly with increasing CCT, from 197.07 to 24.49 in breast meat and from 143.16 to 10.54 in drumstick meat. Breast meat exhibited significantly higher BI values than drumstick meat at every CCT condition (*p* < 0.001).

Total color difference showed a distinct CCT-dependent pattern. Because 6500 K was used as the reference condition, ΔE*ab was necessarily zero at 6500 K for both products. The greatest color differences were observed under the warmest illumination condition, with ΔE*ab values of 42.86 for breast meat and 43.57 for drumstick meat at 2500 K (*p* < 0.001). ΔE*ab progressively declined as CCT approached 6500 K. Above the 6500 K reference, ΔE*ab increased progressively in breast meat, reaching 9.09 at 8500 K (*p* < 0.05). In drumstick meat, ΔE*ab increased to a maximum of 7.61 at 8000 K and subsequently decreased to 6.59 at 8500 K. The mixed-model analysis confirmed significant effects of product type, CCT, and their interaction on ΔE*ab (*p* < 0.001).

### 3.3. Principal Component Analysis

Principal component analysis was conducted using the standardized L*, a*, and b* values from all 442 observations. A single principal component was retained, with an eigenvalue of 2.607, explaining 86.89% of the total variance.

**Figure 4:**
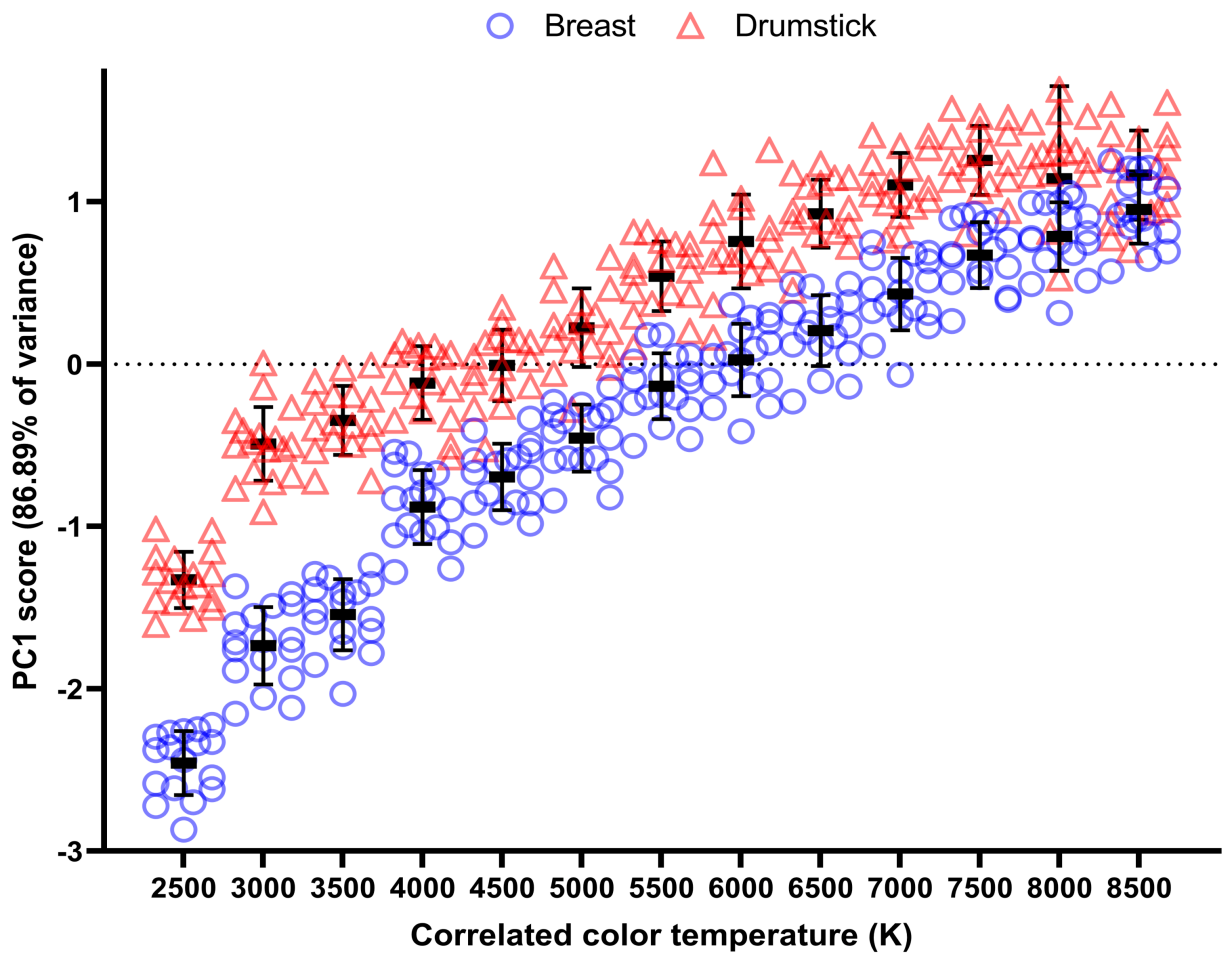
Principal component 1 (PC1) scores of chicken breast and drumstick samples photographed under correlated color temperatures ranging from 2500 to 8500 K. PC1 explained 86.89% of the total variance in the standardized L*, a*, and b* coordinates. Open symbols represent individual biological samples (n = 17 per product at each Correlated color temperature), while black horizontal bars and error bars indicate the mean ± standard deviation. The direction of PC1 scores was reversed for interpretability; therefore, increasing PC1 scores represent increasing L* and decreasing a* and b* values.

The original component loadings were 0.985 for a*, 0.934 for b*, and −0.874 for L*. Thus, PC1 represented a common color dimension in which L* varied in the opposite direction to a* and b*. For graphical presentation, the direction of the PC1 scores was reversed so that increasing PC1 scores represented increasing L* and decreasing a* and b* values. After reversal, the loadings were 0.874 for L*, −0.985 for a*, and −0.934 for b*, so that higher PC1 scores represented higher L* and lower a* and b* values. The distribution of the reversed PC1 scores increased progressively with CCT in both products (Figure 4).

Drumstick samples generally exhibited higher PC1 scores than breast samples at corresponding CCT conditions, whereas the distributions of both products shifted from predominantly negative values under warm illumination to positive values under higher-CCT illumination. This pattern summarized the combined increase in lightness and reduction in redness and yellowness associated with increasing CCT.

## 4. Discussion

The present study demonstrated that correlated color temperature was a major determinant of image-derived color measurements in both skinless chicken breast and skin-on drumstick samples. The Type III fixed-effects analysis supported the individual parameter findings, showing significant effects of sample type, CCT, and their interaction on all primary color coordinates and derived indices, including L*, a*, b*, C*, WI, YI, BI, and ΔE*ab (*p* < 0.001; Table 3). Importantly, the significant product × CCT interactions indicate that breast and skin-on drumstick samples did not respond in parallel to changes in illumination. Therefore, the main effects of product and CCT should not be interpreted as uniform across all conditions; rather, the magnitude and, in some cases, the pattern of the CCT-induced color response depended on the imaged surface. The significant interaction for ΔE*ab similarly demonstrates that the extent of deviation from the 6500 K reference differed between the two sample types. These findings are consistent with previous studies showing that image-derived meat color depends on the interaction among illumination, the imaging system, and the optical properties of the evaluated matrix [4,6,9,10,11,14,15]. However, because the breast samples were skinless while the drumstick samples retained the skin, the observed differences represent the complete optical response of the imaged surfaces, including contributions from skin, subcutaneous fat, and connective tissue, rather than intrinsic muscle-type differences alone. Overall, the mixed-model results demonstrate that a single CCT should not be assumed to produce equivalent color responses across different poultry surfaces without product-specific validation.

An important methodological consideration is that CCT alone does not fully characterize the spectral quality or color-rendering behavior of an LED source. Light sources with similar nominal CCT values may differ substantially in spectral power distribution, color rendering index, fidelity, gamut, and distance from the Planckian locus. Lee and Yoon [18] demonstrated that such differences can alter perceived brightness and visual acceptance even at comparable CCT and illuminance levels. Although their study focused on human-centric office lighting rather than meat imaging, it illustrates that two light sources specified at the same CCT may not produce equivalent spectral stimulation or object-color appearance. Therefore, the 6500 K condition used in the present study should be interpreted as a device-specific operational reference rather than as a universal reference or a physical reproduction of CIE standard illuminant D65. Reproducing comparable imaging conditions across different systems requires characterization and standardization of the light source beyond CCT alone, preferably including spectral power distribution and relevant color rendering metrics.

Lightness showed a clear matrix-dependent response to CCT. In our previous red-meat study [4], L* remained statistically unchanged across the evaluated CCT range, whereas in the present study it was significantly affected by CCT in both breast and skin-on drumstick samples. Breast L* initially decreased slightly and subsequently increased, reaching its highest value at 8500 K, whereas drumstick L* reached its maximum at 7500 K and declined slightly thereafter. The larger L* range observed in breast samples indicates that the recorded lightness of this relatively pale and less pigmented surface was particularly sensitive to changes in illumination. This contrast with the previous red-meat findings supports the conclusion that the effect of CCT on image-derived lightness is matrix-dependent rather than constant across meat types [4]. Girolami et al. [11] similarly found that computer vision produced lower L* values than a colorimeter in beef but higher values in pork and chicken, whereas Tomasevic et al. [10] reported that the direction and magnitude of differences between imaging and conventional color measurements varied according to the physical structure and composition of the evaluated meat product. The contrast between the present findings and our previous red-meat study may reflect fundamental differences in pigment concentration, tissue translucency, light absorption, and scattering [4]. Red meat generally contains more myoglobin and therefore exhibits stronger wavelength-dependent absorption, which may reduce the relative contribution of illumination induced changes to the recorded lightness. In contrast, the paler and less pigmented breast surface may permit a greater contribution from diffuse scattering and from changes in the spectral output of the light source, making image-derived L* more sensitive to CCT. The response of skin-on drumstick samples may additionally reflect scattering and reflection by the skin, subcutaneous fat, and connective tissue. Differences in camera, sensor spectral sensitivity and the fixed white balance and exposure settings may further amplify these matrix-dependent responses. However, because spectral power distribution and sample reflectance were not measured, these mechanisms should be regarded as explanatory hypotheses rather than as directly demonstrated causal relationships.

The practical importance of illumination standardization is further emphasized by the use of L* thresholds for classifying poultry breast meat as dark, normal, or pale. Lee et al. [25] classified breast fillets as dark at L* < 56, normal at L* values between 56 and 62, and pale at L* > 62, and reported that higher L* values were associated with lower pH and water-holding capacity and with greater drip and cooking losses. Similarly, Petracci et al. [26] classified broiler breast fillets as dark at L* < 50, normal at 50 ≤ L* ≤ 56, and pale at L* > 56, and found that pale fillets exhibited lower ultimate pH and greater cooking losses than normal and dark fillets. The different threshold values adopted in these studies also demonstrate that L*-based classification limits are dependent on the biological material, processing conditions, measurement system, and experimental protocol rather than being universally applicable. Notably, changes in CCT in the present study caused the image-derived L* values of the same biological samples to cross numerical boundaries commonly used to distinguish dark, normal, and pale breast meat. Values obtained using computer vision should not be interpreted directly according to thresholds originally established using conventional colorimeters [6,10]. Nevertheless, the observed variation demonstrates that uncontrolled illumination could influence apparent quality classification when image-derived L* values are used in decision-making or predictive models.

Whiteness index exhibited a related, but not identical, response to L*. In breast samples, WI increased markedly with increasing CCT, whereas in skin-on drumstick samples it reached its maximum at 7500 K and remained relatively high thereafter, despite the slight decline in L* at the highest CCT levels (Table 2). Because WI is mathematically derived from L*, a*, and b*, it should not be regarded as an independent measure of lightness. Instead, the increase in WI reflected the combined shift toward greater lightness and lower chromaticity as a* and b* approached zero. In skin-on drumstick samples, this response may also have been influenced by wavelength-dependent absorption and scattering associated with the skin, subcutaneous fat, and connective tissue. Thus, the WI results indicate that higher-CCT illumination produced a lighter and more neutral image-derived appearance rather than simply increasing L*.

Redness (a*) was particularly sensitive to illumination conditions. The a* value decreased markedly with increasing CCT in both sample types, declining from 34.43 to 12.10 in breast samples and from 26.83 to 7.79 in skin-on drumstick samples. Breast samples had higher a* values than drumstick samples at all CCT levels. However, this result should not be interpreted as evidence of intrinsically greater redness in breast muscle because the two sample categories differed not only in anatomical origin but also in skin coverage and overall surface composition. The particularly high a* values observed under warm CCT conditions also reflect the combined influence of the LED spectral output, camera-sensor response, and fixed acquisition settings. Therefore, the recorded a* values should be interpreted as workflow-dependent image outputs rather than as direct measures of intrinsic meat redness or myoglobin-related pigmentation. The decreasing a* pattern was partly consistent with our previous red-meat study, in which a* initially increased up to approximately 5100 K and subsequently declined under higher-CCT illumination [4]. The absence of an initial increase in the present poultry samples further illustrates that the direction and shape of the CCT response cannot be transferred directly between different meat surfaces and imaging workflows.

Previous comparisons of computer vision and conventional colorimetry also demonstrate that image-derived a* is strongly influenced by the interaction between the evaluated surface and the measurement system [6]. Girolami et al. [11] reported substantially higher a* values with computer vision than with a colorimeter in beef, pork, and chicken. Notably, chicken breast had an a* value of 14.97 when evaluated by computer vision but only 0.19 when measured using a colorimeter under 6500 K illumination. Tomasevic et al. [10] similarly found that the magnitude of the difference between computer vision and colorimeter measurements varied considerably among meat products with different compositions and surface structures. Accordingly, image-derived a* should not be considered solely an indicator of pigment concentration. It represents the combined effects of illumination spectrum, wavelength-dependent absorption and scattering, surface composition, camera sensor sensitivity, calibration, and image-processing procedures.

Yellowness-related parameters also showed a pronounced and sample-dependent response to CCT. As CCT increased from 2500 to 8500 K, b* decreased from 52.72 to 8.19 in breast samples and from 47.40 to 1.63 in skin-on drumstick samples (Table 1). YI showed a closely corresponding decline, decreasing from 125.09 to 16.79 in breast samples and from 103.01 to 3.49 in drumstick samples (Table 2). Because YI was calculated from b* relative to L*, these parameters should not be interpreted as statistically or biologically independent responses. Rather, YI expressed the reduction in image-derived yellowness while accounting for concurrent changes in lightness. The increase in breast L* at higher CCTs further contributed to the reduction in YI, whereas the very low b* values obtained for drumstick samples at 8000 and 8500 K resulted in YI values approaching zero.

This pattern contrasted with our previous red-meat study, in which b* generally increased at higher CCTs while L* remained statistically unchanged [4]. Although YI was not calculated in that study, increasing b* combined with stable L* would be expected to increase rather than decrease yellowness-related indices. The opposing responses observed in red meat and poultry demonstrate that CCT does not introduce a constant directional bias in image-derived yellowness. Instead, both the direction and magnitude of the response depend on the evaluated biological surface and imaging system. This interpretation is supported by Girolami et al. [11], who reported substantially higher b* values for chicken breast using computer vision than using a colorimeter under D65 illumination, and by Tomasevic et al. [10], who showed that both the magnitude and direction of b* differences between the two measurement systems varied among meat products with different structures and compositions.

Taken together, the responses of a* and b* demonstrate that image-derived chromatic coordinates cannot be regarded solely as direct indicators of pigment-related redness or yellowness. They represent the combined influence of surface pigmentation, wavelength-dependent absorption and scattering, illumination spectrum, light penetration, sensor response, calibration, and digital image-processing procedures. This interpretation is consistent with Ramanathan et al. [27], who described meat as a complex optical matrix in which heme proteins selectively absorb and reflect specific wavelengths, while tissue structure and water distribution modify light scattering and surface reflectance. Consequently, differences in image-derived a* and b* obtained under different CCT conditions may occur even when the chemical composition and pigment status of the biological sample remain unchanged.

Chroma (C*) decreased markedly with increasing CCT in both sample types, from 63.00 to 14.74 in breast samples and from 54.48 to 8.14 in skin-on drumstick samples. Because C* is calculated from a* and b*, this decline reflected the simultaneous reduction in image-derived redness and yellowness rather than an independent color response. In practical terms, higher-CCT illumination produced a substantially less saturated recorded appearance. This pattern differed from our previous red-meat study, in which C* remained statistically unchanged despite significant CCT-related variation in a* and b* [4]. Previous comparisons between computer vision and conventional colorimetry have also shown that C* values can differ substantially according to the measurement system and the physical structure of the evaluated meat matrix [6,10,11]. The present results therefore indicate that the effect of CCT on apparent color saturation is surface-dependent.

Browning index also decreased markedly with increasing CCT, from 197.07 to 24.49 in breast samples and from 143.16 to 10.54 in skin-on drumstick samples. However, BI is mathematically derived from L*, a*, and b* and should not be interpreted as direct evidence of chemical browning, oxidative deterioration, or pigment oxidation. Its decline primarily reflected the pronounced reductions in a* and b*, together with concurrent changes in L*. Accordingly, BI should be interpreted here as a composite image-derived color index rather than as a direct biochemical measurement of browning. Similarly, the higher BI values observed in breast samples describe the colorimetric response of the complete imaged surface under the applied illumination conditions and do not necessarily indicate a greater degree of actual browning.

The ΔE*ab results demonstrated the practical magnitude of the CCT effect. Relative to the 6500 K reference, the greatest total color differences were observed at 2500 K, reaching 42.86 in breast samples and 43.57 in skin-on drumstick samples. These differences substantially exceeded commonly used criteria for visually perceptible color differences, indicating that warm illumination markedly altered the recorded appearance of the same biological samples [10]. Above the reference condition, ΔE*ab values were smaller but remained visually relevant, reaching 9.09 at 8500 K in breast samples and a maximum of 7.61 at 8000 K in drumstick samples. A visually detectable total color difference between contrasting CCT conditions was also reported in our previous red-meat investigation [4]. Therefore, the effect of illumination was not limited to extremely warm CCTs; substantial deviations from the reference appearance also remained under higher-CCT conditions. These findings confirm that illumination temperature can introduce large systematic differences in image-derived color, although the magnitude and pattern of this response depend on the evaluated surface and imaging protocol. They also indicate that selecting 6500 K as a nominal reference does not eliminate illumination-related variability unless the complete spectral and imaging conditions are reproduced consistently.

The practical significance of these illumination-induced differences may also extend to visual perception, although instrumental and sensory color responses should not be assumed to be equivalent. A color difference of ΔE = 5 is generally easily noticeable to the human eye, especially when the two colors are viewed side by side. In common practical classifications, a ΔE of 1–2 is barely noticeable, while differences above 3 can usually be seen by most people; a value around 5 appears quite distinct [16]. Corlett et al. [28] reported that consumer perception of meat redness differed according to muscle type, storage period, and retail display time, whereas its correlation with an instrumental oxymyoglobin-to-metmyoglobin reflectance measure was relatively low. This finding indicates that objective color measurements capture only part of the visual information contributing to consumer assessment. Accordingly, the substantial CCT-induced color differences observed in the present study may alter the perceived appearance of poultry surfaces; however, sensory evaluation would be required to determine the extent to which these image-derived changes influence consumer perception.

Principal component analysis provided a multivariate summary of the coordinated changes in the primary CIELAB parameters. PC1 had an eigenvalue of 2.607 and explained 86.89% of the total variance in the standardized L*, a*, and b* data. In the original PCA solution, PC1 was positively associated with a* and b* and negatively associated with L*, with loadings of 0.985, 0.934, and −0.874, respectively (Figure 4). Thus, most of the observed color variation followed a common dimension characterized by simultaneous changes in lightness, redness, and yellowness. This high proportion of explained variance indicates that, within the present dataset and imaging workflow, the image-derived chicken meat color space was dominated by a nearly one-dimensional CCT-related gradient rather than by independent variation along the three CIELAB coordinates. The direction of the PC1 scores was subsequently reversed solely to facilitate interpretation. After this transformation, PC1 increased progressively with CCT in both sample types, representing an overall transition from a darker and more chromatic recorded appearance under warm illumination to a lighter and less chromatic appearance under higher CCT illumination.

The PCA findings therefore support the univariate results by showing that CCT did not alter each color coordinate in isolation; instead, it produced a coordinated shift in the overall image-derived color profile. The generally higher transformed PC1 scores of skin-on drumstick samples should not, however, be interpreted solely as an intrinsic muscle difference because skin, subcutaneous fat, and connective tissue contributed to the optical response of the imaged surface. Furthermore, PCA was used as a descriptive dimensionality-reduction technique and did not account for the repeated-measures structure of the experiment. Statistical inference regarding sample type, CCT, and their interaction was therefore based on the mixed-model analysis presented in Table 3 rather than on the visual separation or distribution of PCA scores.

From a practical perspective, the present results demonstrate that CCT should be treated as a critical acquisition variable in image-based poultry color analysis. Images acquired under different CCT conditions cannot be compared reliably without strict standardization of illumination, even when exposure, camera position, imaging distance, sample position, and viewing geometry are kept constant. Standardization should therefore include not only the CCT but also the other IPS setup. The need for comprehensive standardization is also supported by Brugiapaglia and Destefanis [9], who evaluated the use of photographs for sensory assessment of meat color. They emphasized that lighting, background, and camera settings must be standardized to obtain a faithful reproduction of meat appearance and recommended the use of a color target to verify color accuracy. Although their study employed a single illumination condition and did not directly compare different CCT levels, it demonstrates that the validity of photograph-based color evaluation depends on the control of the complete image-acquisition environment rather than on camera calibration alone.

Although the present findings demonstrate the importance of controlling illumination conditions in image-based meat color assessment, the light sources were characterized primarily according to correlated color temperature, without direct spectroradiometric measurements. Future studies should therefore incorporate spectral power distribution measurements and relevant color-rendering metrics to determine more precisely how individual wavelength components influence image-derived color values. Such characterization would also facilitate the reproducibility and comparison of imaging systems using light sources with similar CCT values but potentially different spectral properties.

## 5. Conclusions

Overall, the present findings demonstrate that CCT is a major source of systematic variation in image-derived poultry color measurements. These results indicate that CCT must be strictly controlled and reported in image-based poultry color analysis. Its effect extended across the primary CIELAB coordinates and all evaluated derived indices, while the significant sample type × CCT interactions showed that the response was specific to the imaged surface. Warm illumination produced the greatest deviation from the 6500 K reference, but visually relevant differences also remained under higher-CCT conditions. Therefore, 6500 K may serve as a practical reference condition only when the complete illumination and imaging protocol is strictly standardized, reproducibly maintained, and validated for the specific poultry surface being evaluated.

## Figures and Tables

**Table 1:** CIELAB color parameters of chicken breast and drumstick meat collected under different correlated color temperature (CCT) conditions.

CCT (K)	Product	L*	a*	b*	C*
2500	Breast	60.21 ± 1.40 ^aB^	34.43 ± 1.73 ^aA^	52.72 ± 2.04 ^aA^	63.00 ± 1.55 ^aA^
	Drumstick	65.74 ± 1.15 ^abcA^	26.83 ± 1.46 ^aB^	47.40 ± 1.57 ^aB^	54.48 ± 1.55 ^aB^
3000	Breast	59.05 ± 1.67 ^aB^	28.08 ± 1.99 ^bA^	34.97 ± 2.32 ^bA^	44.92 ± 1.57 ^bA^
	Drumstick	65.47 ± 1.58 ^bcA^	21.04 ± 1.46 ^bB^	27.53 ± 1.53 ^bB^	34.68 ± 1.61 ^bB^
3500	Breast	59.14 ± 1.59 ^aB^	26.53 ± 1.80 ^cA^	31.52 ± 2.36 ^cA^	41.27 ± 1.58 ^cA^
	Drumstick	65.16 ± 1.51 ^cA^	19.72 ± 1.37 ^bB^	23.75 ± 1.61 ^cB^	30.90 ± 1.59 ^cB^
4000	Breast	63.90 ± 1.52 ^bB^	23.80 ± 1.81 ^dA^	30.30 ± 2.22 ^cA^	38.60 ± 1.61 ^dA^
	Drumstick	65.48 ± 1.40 ^bcA^	18.16 ± 1.42 ^cB^	19.72 ± 1.52 ^dB^	26.84 ± 1.64 ^dB^
4500	Breast	63.95 ± 1.37 ^bA^	22.37 ± 1.69 ^eA^	26.64 ± 2.21 ^dA^	34.85 ± 1.60 ^eA^
	Drumstick	64.92 ± 1.64 ^cA^	16.79 ± 1.34 ^dB^	16.48 ± 1.47 ^eB^	23.56 ± 1.52 ^eB^
5000	Breast	64.38 ± 1.36 ^bcA^	20.54 ± 1.59 ^fA^	23.20 ± 2.14 ^eA^	31.05 ± 1.65 ^fA^
	Drumstick	65.61 ± 1.79 ^bcA^	15.40 ± 1.35 ^eB^	13.36 ± 1.48 ^fB^	20.43 ± 1.48 ^fB^
5500	Breast	65.51 ± 1.27 ^bcdB^	18.53 ± 1.54 ^gA^	19.77 ± 2.09 ^fA^	27.17 ± 1.66 ^gA^
	Drumstick	67.16 ± 1.49 ^abdA^	13.96 ± 1.29 ^fB^	10.39 ± 1.44 ^gB^	17.45 ± 1.36 ^gB^
6000	Breast	65.62 ± 1.50 ^bcdB^	17.09 ± 1.51 ^hA^	17.18 ± 1.98 ^gA^	24.31 ± 1.58 ^hA^
	Drumstick	67.96 ± 2.48 ^defA^	12.86 ± 1.37 ^fB^	7.73 ± 1.59 ^hB^	15.08 ± 1.43 ^hB^
6500	Breast	66.02 ± 1.43 ^cdB^	15.76 ± 1.53 ^iA^	14.77 ± 1.92 ^hA^	21.68 ± 1.51 ^iA^
	Drumstick	68.64 ± 1.61 ^defA^	11.51 ± 1.17 ^gB^	6.74 ± 1.52 ^hB^	13.42 ± 1.20 ^iB^
7000	Breast	66.95 ± 1.53 ^deB^	14.49 ± 1.52 ^ijA^	12.59 ± 1.86 ^iA^	19.29 ± 1.38 ^jA^
	Drumstick	69.10 ± 1.48 ^efA^	10.39 ± 1.18 ^ghB^	4.26 ± 1.57 ^iB^	11.33 ± 1.22 ^jB^
7500	Breast	68.30 ± 1.31 ^efA^	13.50 ± 1.58 ^jkA^	10.78 ± 1.85 ^jA^	17.39 ± 1.33 ^kA^
	Drumstick	69.52 ± 1.49 ^eA^	9.32 ± 1.24 ^hiB^	2.41 ± 1.61 ^jB^	9.74 ± 1.30 ^kB^
8000	Breast	68.83 ± 1.38 ^fA^	12.93 ± 1.61 ^kmA^	9.64 ± 1.81 ^jkA^	16.25 ± 1.31 ^kA^
	Drumstick	67.67 ± 5.14 ^dfA^	8.73 ± 1.29 ^ijB^	0.97 ± 1.51 ^jB^	8.90 ± 1.28 ^kmB^
8500	Breast	69.63 ± 1.43 ^fA^	12.10 ± 1.63 ^mA^	8.19 ± 1.75 ^kA^	14.74 ± 1.34 ^mA^
	Drumstick	67.53 ± 1.99 ^adfB^	7.79 ± 1.23 ^jB^	1.63 ± 1.74 ^jB^	8.14 ± 1.20 ^mB^

L*: lightness; a*: red-green position; b*: yellow-blue position; C*: chroma; K: Kelvin. Values are presented as arithmetic mean ± standard deviation (n = 17). Within the same product and color parameter, mean values with different lowercase superscript letters are significantly different among CCT conditions (*p* < 0.05). Within the same CCT condition and color parameter, mean values with different uppercase superscript letters are significantly different between breast and drumstick meat (*p* < 0.05). Superscript letters were assigned based on Sidak-adjusted pairwise comparisons of the estimated marginal means derived from the linear mixed model.

**Table 2:** Derived color indices and total color difference in chicken breast and drumstick meat under different correlated color temperature (CCT) conditions.

CCT (K)	Product	WI	YI	BI	ΔE*ab
2500	Breast	25.46 ± 1.33 ^aB^	125.09 ± 3.47 ^aA^	197.07 ± 8.14 ^aA^	42.86 ± 2.21 ^aA^
	Drumstick	35.63 ± 1.48 ^aA^	103.01 ± 3.51 ^aB^	143.16 ± 6.89 ^aB^	43.57 ± 0.65 ^aA^
3000	Breast	39.19 ± 1.58 ^bB^	84.58 ± 4.37 ^bA^	118.27 ± 6.47 ^bA^	24.70 ± 0.67 ^bA^
	Drumstick	51.05 ± 1.89 ^bA^	60.11 ± 3.70 ^bB^	76.15 ± 5.31 ^bB^	23.13 ± 0.44 ^bB^
3500	Breast	41.91 ± 1.54 ^cB^	76.09 ± 4.70 ^cA^	104.67 ± 6.22 ^cA^	21.45 ± 2.16 ^cA^
	Drumstick	53.41 ± 1.74 ^cA^	52.08 ± 3.57 ^cB^	65.93 ± 4.70 ^cB^	19.23 ± 0.43 ^cB^
4000	Breast	47.14 ± 1.68 ^dB^	67.71 ± 4.45 ^dA^	88.76 ± 5.72 ^dA^	18.05 ± 2.20 ^dA^
	Drumstick	56.27 ± 1.92 ^dA^	43.06 ± 3.61 ^dB^	54.90 ± 4.74 ^dB^	14.97 ± 0.53 ^dB^
4500	Breast	49.84 ± 1.56 ^eB^	59.48 ± 4.49 ^eA^	77.40 ± 5.20 ^eA^	14.26 ± 2.22 ^eA^
	Drumstick	57.72 ± 1.80 ^dA^	36.28 ± 3.22 ^eB^	47.09 ± 3.91 ^eB^	11.73 ± 0.41 ^eB^
5000	Breast	52.72 ± 1.64 ^fB^	51.48 ± 4.45 ^fA^	66.44 ± 5.01 ^fA^	10.51 ± 2.22 ^fA^
	Drumstick	59.99 ± 1.99 ^eA^	29.10 ± 3.27 ^fB^	38.95 ± 3.75 ^fB^	8.31 ± 0.54 ^fB^
5500	Breast	56.08 ± 1.67 ^gB^	43.11 ± 4.40 ^gA^	55.35 ± 4.64 ^gA^	6.72 ± 1.45 ^gA^
	Drumstick	62.80 ± 1.77 ^fA^	22.13 ± 3.14 ^gB^	31.14 ± 3.25 ^gB^	4.74 ± 0.56 ^ghB^
6000	Breast	57.88 ± 1.80 ^ghB^	37.41 ± 4.19 ^hA^	48.30 ± 4.32 ^hA^	4.71 ± 2.07 ^hiA^
	Drumstick	64.56 ± 2.47 ^fgA^	16.24 ± 3.20 ^hB^	25.13 ± 3.12 ^hB^	4.25 ± 2.14 ^hiA^
6500	Breast	59.68 ± 1.77 ^hB^	31.96 ± 4.09 ^iA^	41.78 ± 3.96 ^iA^	Reference
	Drumstick	65.88 ± 1.75 ^ghA^	14.02 ± 3.09 ^hB^	21.90 ± 2.70 ^hB^	Reference
7000	Breast	61.72 ± 1.79 ^iB^	26.86 ± 3.89 ^jA^	35.74 ± 3.49 ^jA^	4.52 ± 1.26 ^iA^
	Drumstick	67.08 ± 1.63 ^hiA^	8.81 ± 3.21 ^iB^	16.76 ± 2.72 ^iB^	2.88 ± 0.26 ^iB^
7500	Breast	63.84 ± 1.63 ^jB^	22.53 ± 3.79 ^kA^	30.79 ± 3.14 ^kA^	6.21 ± 2.30 ^ghA^
	Drumstick	67.98 ± 1.70 ^iA^	4.95 ± 3.28 ^jB^	12.84 ± 2.90 ^jB^	5.05 ± 0.37 ^ghB^
8000	Breast	64.83 ± 1.66 ^jkB^	19.99 ± 3.64 ^kA^	28.03 ± 2.92 ^klA^	7.41 ± 1.92 ^gA^
	Drumstick	66.45 ± 5.17 ^ghiA^	2.13 ± 3.28 ^jB^	10.59 ± 3.51 ^jB^	7.61 ± 3.20 ^fA^
8500	Breast	66.23 ± 1.69 ^kA^	16.79 ± 3.48 ^lA^	24.49 ± 2.73 ^lA^	9.09 ± 2.31 ^fA^
	Drumstick	66.52 ± 2.17 ^hiA^	3.49 ± 3.67 ^jB^	10.54 ± 3.07 ^jB^	6.59 ± 0.71 ^fgB^

WI: Whiteness index; YI: Yellowness index; BI: Browning index; ΔE*ab: Total color difference; K: kelvin. Values are presented as arithmetic mean ± standard deviation (n = 17). Within the same product and color parameter, mean values with different lowercase superscript letters are significantly different among CCT conditions (*p* < 0.05). Within the same CCT condition and color parameter, mean values with different uppercase superscript letters are significantly different between breast and drumstick meat (*p* < 0.05). Superscript letters were assigned based on Sidak-adjusted pairwise comparisons of the estimated marginal means derived from the linear mixed model. For ΔE*ab, the 6500 K condition was used as the reference and was excluded from inferential analysis because its value was necessarily zero for every sample.

**Table 3:** Type III tests of fixed effects for product type, correlated color temperature, and their interaction on image-derived color parameters and derived indices.

Parameter	Product		CCT		Product × CCT	
	F (df_1_, df_2_)	*p*	F (df_1_, df_2_)	*p*	F (df_1_, df_2_)	*p*
L*	36.097 (1, 32.0)	<0.001	74.895 (12, 213.1)	<0.001	21.346 (12, 213.1)	<0.001
a*	220.102 (1, 31.9)	<0.001	899.377 (12, 212.8)	<0.001	8.911 (12, 212.8)	<0.001
b*	396.526 (1, 31.9)	<0.001	2678.266 (12, 212.5)	<0.001	9.082 (12, 212.5)	<0.001
C*	670.501 (1, 32.0)	<0.001	4186.437 (12, 209.0)	<0.001	14.058 (12, 209.0)	<0.001
WI	238.690 (1, 32.0)	<0.001	1325.991 (12, 211.5)	<0.001	35.793 (12, 211.5)	<0.001
YI	506.542 (1, 31.9)	<0.001	3512.525 (12, 214.3)	<0.001	11.201 (12, 214.3)	<0.001
BI	613.070 (1, 31.9)	<0.001	4450.684 (12, 200.6)	<0.001	77.855 (12, 200.6)	<0.001
ΔE*ab	40.585 (1, 76.7)	<0.001	1240.531 (11, 235.4)	<0.001	5.128 (11, 235.4)	<0.001

L*: lightness; a*: red-green position; b*: yellow-blue position; C*: chroma; WI: Whiteness index; YI: Yellowness index; BI: Browning index; ΔE*ab: Total color difference; K: kelvin. Denominator degrees of freedom were estimated using the Satterthwaite approximation. For ΔE*ab, the 6500 K reference condition was excluded from inferential analysis. Values are presented as F statistics with numerator and denominator degrees of freedom in parentheses.

## Data Availability

The original contributions presented in this study are included in the article/Supplementary Materials. Further inquiries can be directed to the corresponding author.

## Supplementary Materials

The following supporting information can be downloaded at: https://www.mdpi.com/article/10.3390/foods15152602/s1.

## Abbreviations

The following abbreviations are used in this manuscript:

AR(1)	First-order autoregressive covariance structure
BGGR	Blue–Green–Green–Red Bayer pattern
CCT	Correlated color temperature
CIELAB	Commission Internationale de l’Éclairage L*a*b* color space
CMOS	Complementary metal-oxide-semiconductor
DIY	Do-it-yourself
DNG	Digital Negative
EV	Exposure value
IPS	Image processing system
ROI	Region of interest
TIFF	Tagged Image File Format
